# A Novel Method for Determining Angular Speed and Acceleration Using Sin-Cos Encoders

**DOI:** 10.3390/s21020577

**Published:** 2021-01-15

**Authors:** Manuel Alcázar Vargas, Javier Pérez Fernández, Juan M. Velasco García, Juan A. Cabrera Carrillo, Juan J. Castillo Aguilar

**Affiliations:** Department of Mechanical Engineering, University of Málaga, 29071 Málaga, Spain; manuel.alcazar@uma.es (M.A.V.); javierperez@uma.es (J.P.F.); juanmav@uma.es (J.M.V.G.); jcabrera@uma.es (J.A.C.C.)

**Keywords:** Sin-Cos encoder, ABS encoder, wheel angular speed, Savitzky-Golay filter, vehicle control systems, vehicle sensors

## Abstract

The performance of vehicle safety systems depends very much on the accuracy of the signals coming from vehicle sensors. Among them, the wheel speed is of vital importance. This paper describes a new method to obtain the wheel speed by using Sin-Cos encoders. The methodology is based on the use of the Savitzky–Golay filters to optimally determine the coefficients of the polynomials that best fit the measured signals and their time derivatives. The whole process requires a low computational cost, which makes it suitable for real-time applications. This way it is possible to provide the safety system with an accurate measurement of both the angular speed and acceleration of the wheels. The proposed method has been compared to other conventional approaches. The results obtained in simulations and real tests show the superior performance of the proposed method, particularly for medium and low wheel angular speeds.

## 1. Introduction

The proper determination of the angular speed of vehicle wheels is of particular importance for active safety systems: Anti-lock Braking Systems (ABS), Traction Control System (TCS), Electronic Stability Program (ESP), etc. [1]. The information from the wheel speed sensors is used as an input parameter to traction and braking control systems [2,3,4]. Tire longitudinal traction and braking forces depend essentially on the slip ratio, which is the relationship between wheel peripherical speed and the linear speed of the vehicle [5,6,7]. In the field of brake control systems, it is crucial to determine the angular speed of the wheels in medium and low-speed conditions [6,8,9]. This is because ABS prevents the wheels from locking, so it is required to determine whether the wheel is moving slowly or if it is already locked.

Currently, most of the sensors used to determine the angular speed of the wheels are of the incremental encoder type [1,9]. These provide one or more rectangular signals whose frequency increases as does the angular speed of the wheel. Basically, there are three methods: *M-type*, *T-type*, and a combination of both: *MT-type* to process the output of wheel sensors to estimate the angular speed of the wheels [10]. The first one is more appropriate for high speeds. The computational cost of this method is very low which makes it suitable for real-time systems. However, the main drawback is that the measurement error is high at low speeds. On the contrary, the second one provides highly accurate measurements at low speed but the performance is poor at high speeds. In addition, the computational cost is higher because it is required to perform a division [11]. The third method is based on a combination of the other two. It provides better measurements at medium-low speed, but the computational cost is also higher.

Consequently, all three methods mentioned above have drawbacks that make them inappropriate for some conditions. The main problem is related to the fact that they are based on the use of pulses. When the speed is medium or low, the number of pulses per unit of time can be low or null. This way, none of the previous methods can update the estimated velocity unless a pulse is detected. However, as far as braking control algorithms are concerned, it is of vital importance to make use of reliable wheel angular speed and acceleration measurements. Several modifications of the M/T method have been proposed to cope with the previously mentioned drawbacks. The goals of these proposals are varied, but, in general, they try to improve accuracy and/or computational cost. Thus, the time stamping concept was used to capture the encoder transitions and their time instants at a high clock frequency in [12]. These data were used to estimate position, velocity, and acceleration by means of polynomial interpolation of the encoder events and polynomial extrapolation at the time of interest. Experiments showed improvement of the velocity and acceleration estimations. Similarly, a method to estimate angular velocity by removing the periodical disturbances introduced by sensor imperfections was presented in [13]. To do so, a number of harmonic components of the disturbance were identified and used to improve the velocity estimation. The authors claimed that the proposed scheme represented an improvement over the time stamping algorithm since better estimates were obtained without using a large number of events or introducing a long delay. Similarly, a Single-Phase Self-Adaptative M/T method was proposed in [14] to suppress measurement error and extend speed measurement. The use of a compensation routine was proposed to reduce measurement errors and bias in [15]. These methods improved the estimation accuracy but they required a higher computational cost compared to the conventional M/T method. On the contrary, improvements of the M/T were described in [16,17,18] to reduce the computational cost by avoiding performing the division operations that the conventional M/T method requires. This way, a so-called Divisionless MT-type velocity estimation algorithm was proposed. The main advantage of this method was that it only required addition and multiplication operations, allowing a cost-effective implementation. Finally, the use of a Kalman filter [19,20] was proposed to increase the accuracy of the calculation of the wheel angular speed and acceleration in ABS systems by removing measurement noise and improving the differentiation method.

Another type of sensor used to measure absolute mechanical angles are resolvers. These devices are abundantly used in industrial applications thanks to their high accuracy, resolution and robustness [21]. Resolvers produce quadrature sinusoidal electrical signals representative of the angular position of the shaft. These signals have to be processed with a suitable converter to determine the angular position. On one hand, open-loop conversion methods have been proposed in the literature to carry out this task. Thus, linearization-based techniques, in which the sinusoidal signals were converted into a linear output signal from which displacement can be determined, were described in [22,23,24]. Trigonometric methods yield the angular position directly and have a good dynamic response. However, they generally resort to lookup tables to perform the arctangent computation, which increases memory requirements. In addition, differentiation and filtering to remove high frequency noise, which cause phase delay, are required to obtain angular speed and acceleration [25,26,27]. Further less-common open-loop methods can be found in [28,29,30,31].

On the other hand, most closed-loop converters are based on the use of the Phase-Locked Loop (PLL) technique [32,33,34]. These methods track the angular position and speed smoothly and accurately, but the filter gains have to be properly tuned and it may have problems for high angular acceleration rates. Kalman filter-based closed-loop converters have also been described in [35].

With the massive introduction of electric vehicles, more and more permanent magnet synchronous motors (PMSM) are being used. These electric motors make use of Sin-Cos type encoders. These encoders allow the determination of the relative position between a rotor and a stator with high accuracy, which is decisive for the control of this type of motors. They provide two analog outputs: the sine and the cosine of the relative angle between the rotor and the stator [36,37]. Commonly, the operation principles of resolver converters are applicable to Sin-Cos type encoders. Hence, open-loop and closed-loop converters can be used to provide the angular position from signals provided by this type of sensor.

Regarding the estimation of angular acceleration from resolvers and Sin-Cos encoders, to the best of our knowledge, only modifications of the PLL described above are found in literature, building another loop at the output of the speed estimator to obtain angular acceleration [38,39]. These systems must be tuned for each application, and it is necessary to consider the bandwidth. In commercial chips, this adjustment is usually done in an analogical way, since they consist of capacitors and resistors [40,41]. 

On the other hand, when the speed of the rotating element equipped with Sin-Cos encoders is high, counting zero-crossings gives a sufficiently approximate measurement of the angular speed. However, in the automotive field, and more specifically within traction control and braking algorithms, it is interesting to measure low angular speeds. Therefore, it is necessary to address this problem to find an appropriate strategy for all wheel speed conditions.

In this work, a novel open-loop method for the determination of speed and angular acceleration from the signals provided by a Sin-Cos encoder is proposed. The proposed approach is based on the use of Savitzky-Golay (S-G) filters. The S-G filter has been described to be used in chemical analysis, image processing fields, signal processing and biomedical data processing [42,43,44]. However, to the best of our knowledge, the use of this method with Sin-Cos encoders and resolvers has not been proposed up to now. 

The main advantages of this method are related to a fast and direct calculation of time derivatives, its low computational cost, and that the delay introduced in the filtered signal is known in advance. A further advantage of the proposed method is that the matrix defined in the calculations is composed of only integer numbers. In addition, the mathematical operations required to obtain the filtered data involve mainly integer numbers. Noninteger numbers are only required in the last stage of the filter. Finally, a remarkable advantage of the developed method is that angular speed and acceleration can be obtained as continuous signals by using only additions and multiplications. These advantages simplify the implementation of this method in FPGA-based systems since less computational effort and fewer logic blocks are required. Thus, it properly measures high, medium, and very low speeds. On the other hand, the main drawback is that the order and number of side-points of the filter have to be properly determined. The method developed for the determination of speed and angular acceleration is described. The performance of the proposed method has been evaluated by means of simulations. Finally, a validation of the method from experimental data has been carried out. In automotive applications, wheel angular speeds can be low in some cases (i.e., traction and braking control systems). This way, the use of these sensors in low-speed conditions requires new methods to provide accurate measurements. This paper is intended to serve as an approach to the use of S-G filters in this kind of applications.

This work is structured as follows: first, the mathematical development of the algorithm presented in the work is exposed in Section 2. Next, the results obtained in simulations and real tests are included in Section 3. Results are divided into two subsections. In the first one, the method is validated and its performance is verified by means of simulations. In the second one, the algorithm developed is implemented in a real system and its performance is evaluated. The advantages of the proposed method and the conclusions of this work and are drawn in Section 4.

## 2. Materials and Methods

Sin-Cos encoders provide two analog signals: the sine and the cosine of the relative angle between the rotor and the stator [36,37]. The following novel method is proposed to determine the rotor angular speed and acceleration, which is the fundamental contribution of this work. This method resorts to the Savitzky-Golay filter to calculate the time derivatives of the output signals. This way, the angular speed and acceleration are computed requiring only sums and multiplications. The mathematics of this method is described next. 

Be the encoder outputs *x* and *y* (Equations (1) and (2)):

(1) x=Acos(ωt+φ),

(2) y=Asin(ωt+φ),

where A is the amplitude, *ω* is the angular speed, and φ is the phase shift. The relative angle between the rotor and the stator is given by expression Equation (3):

(3) $$\theta =\mathrm{atan}\left(\frac{y}{x}\right),$$

The angular speed of the rotor is given by the time derivative of the angular coordinate Equation (4):

(4) $$\frac{d\theta }{dt}=\dot{\theta }=\frac{\frac{d}{dt}\left(\frac{y}{x}\right)}{1+{\left(\frac{y}{x}\right)}^{2}},$$

Operating:

(5) $$\dot{\theta }=\frac{\frac{x\dot{y}-\dot{x}y}{x^{2}}}{\frac{x^{2}+y^{2}}{x^{2}}},$$

(6) $$\dot{\theta }=\frac{x\dot{y}-\dot{x}y}{x^{2}+y^{2}},$$

where $x^{2}+y^{2}=A^{2}\sin^{2}\left(\omega t+\varphi \right)+A^{2}\cos^{2}\left(\omega t+\varphi \right)=A^{2}$, is constant. It is rewritten:

(7) $$\dot{\theta }=\frac{1}{A^{2}}\left(x\dot{y}-\dot{x}y\right),$$

Differentiating again with respect to time and rearranging, the expression for angular acceleration can be obtained Equation (8):

(8) $$\frac{d^{2}\theta }{dt^{2}}=\ddot{\theta }=\frac{1}{A^{2}}\left(x\ddot{y}-\ddot{x}y\right),$$

Therefore, it is necessary to differentiate the sensor signals to obtain both the angular rotor speed and acceleration. To do so, the Savitzky-Golay (SG) filters [45,46] are used to filter the signals and determine their time derivatives. These filters optimally fit a set of data points to polynomials of different orders. In order to use this tool, the points have to be equispaced in time and cannot have discontinuities. Some of the advantages of this method include the following:

- It allows the direct calculation of the time derivatives.
- The delay of all time derivatives, including the zeroth derivative is identical.
- Once the filter parameters are determined, the computational cost is very small since only additions and multiplications are required. This allows it to be used in real-time applications with very high sampling and processing frequencies.
- The Savitzky-Golay filter optimally fits a set of data points to a polynomial using least-squares regression.

To use the SG filter, it is necessary to set the order of the interpolation polynomial and the number of side points. Once defined, the matrix of coefficients **g** is calculated. Each of the columns of this matrix constitutes the convolution vector used to determine the time derivatives. The way to proceed with the mathematics is different if the angular speed is calculated in real-time or if it is obtained through postprocessing computation. Thus, in real-time, the convolution operation is replaced by the dot product of the corresponding column of the matrix **g** by the last *m* elements of the data vector (**x**), where *m* is the number of rows of the matrix **g** (*m* = 2 · *sidepoints* + 1). In this case, the delay of the output signal is equal to the number of side points.

On the contrary, for data postprocessing applications, a single convolution of the whole data vector and the corresponding column of the matrix **g** is performed. In the latter case, the signal is not delayed.

This way, and for real-time applications such as braking control systems, the filtered value of the signal for the point *k* ($\hat{x_{k}}$) is the dot product of the first column of the convolution matrix (**g**_0_) by the last values of the data vector (**x**) by the term (−Δ*t*)^−*p*^ and by *p*!, where *p* is the order of the time derivative (see Equations (9)–(11)).

(9) $$\hat{x_{k}}=0!\cdot g_{0}{}^{T}\cdot x\cdot {\left(-\Delta t\right)}^{-0}=g_{0}{}^{T}\cdot x$$

(10) $$\hat{\dot{x_{k}}}=1!\cdot g_{1}{}^{T}\cdot x\cdot {\left(-\Delta t\right)}^{-1}=-g_{1}{}^{T}\cdot x\cdot \Delta t^{-1}$$

(11) $$\hat{\ddot{x_{k}}}=2!\cdot g_{2}{}^{T}\cdot x\cdot {\left(-\Delta t\right)}^{-2}=2\cdot g_{2}{}^{T}\cdot x\cdot \Delta t^{-2}$$

**Figure 1:**
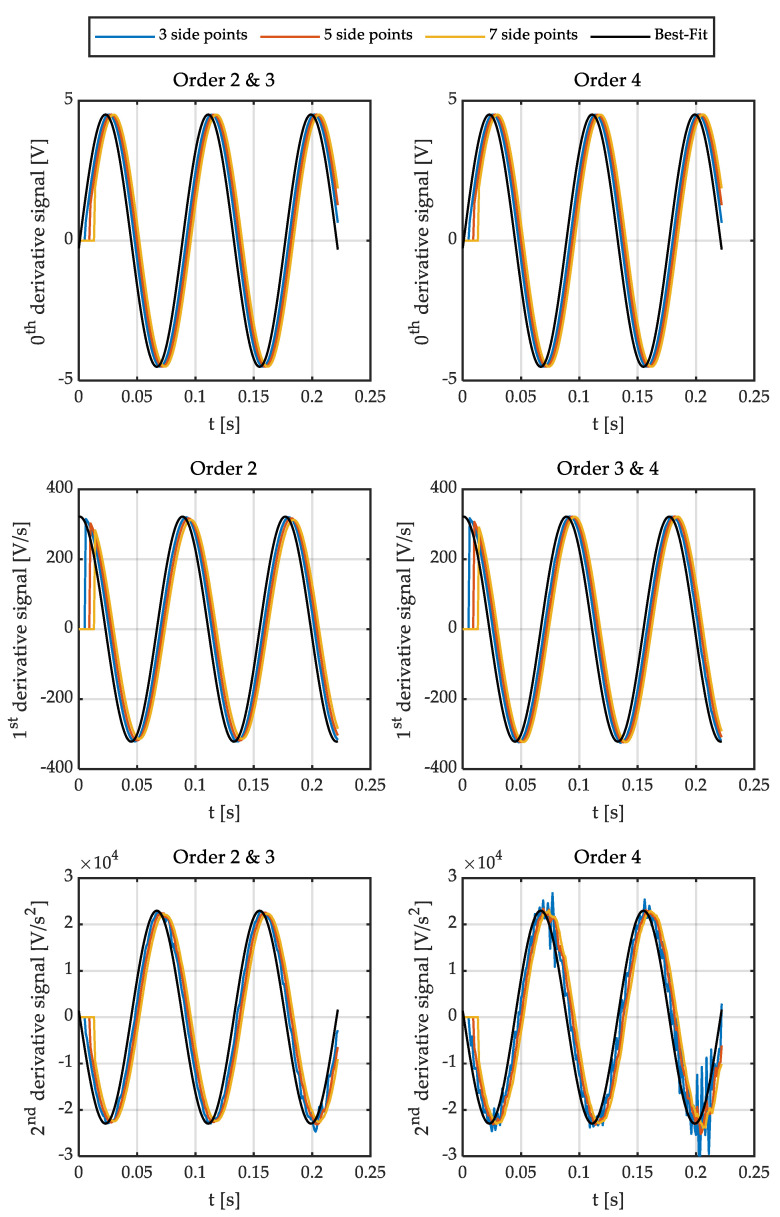
Signals fitted by a sine (black) and signals fitted by polynomials using the Savitzky-Golay method. (**top**) zeroth derivative. (**center**) first derivative. (**bottom**) second derivative.

**Figure 2:**
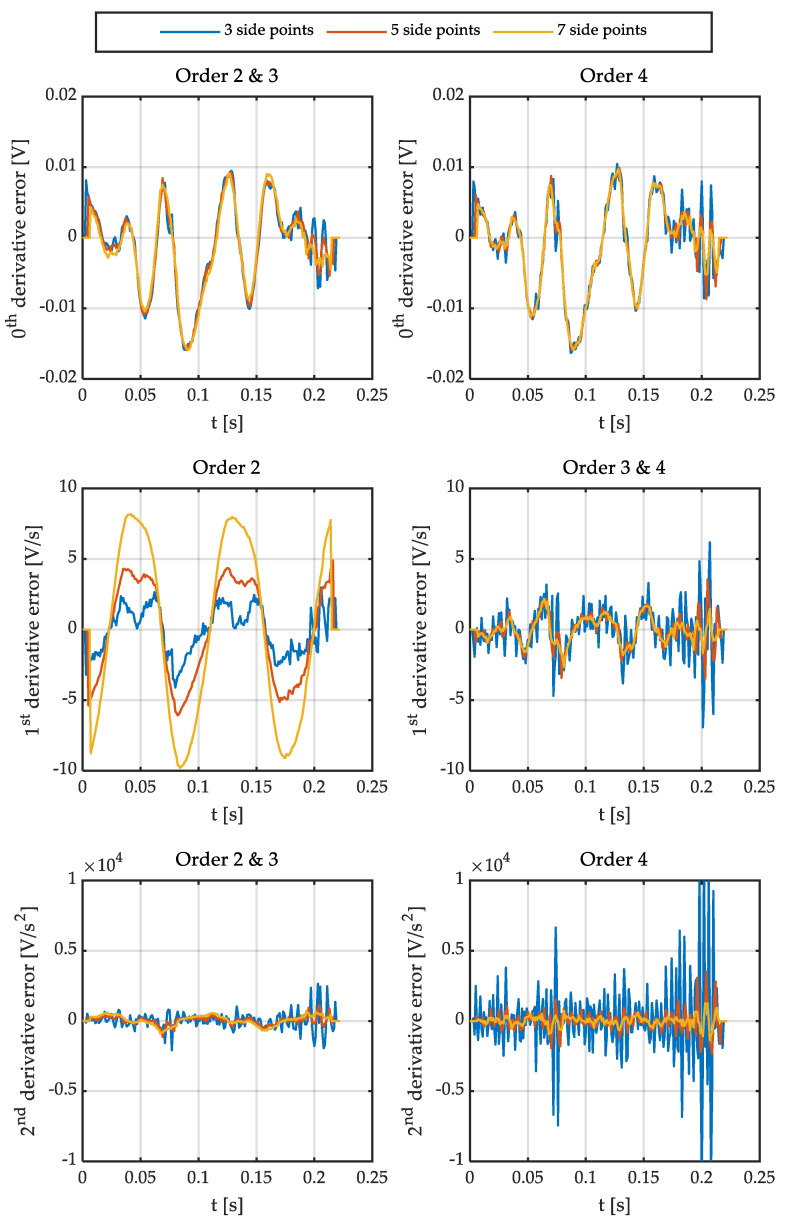
Errors of the signals fitted by polynomials using the Savitzky-Golay method. (**top**) zeroth derivative. (**center**) first derivative. (**bottom**) second derivative.

**Figure 3:**
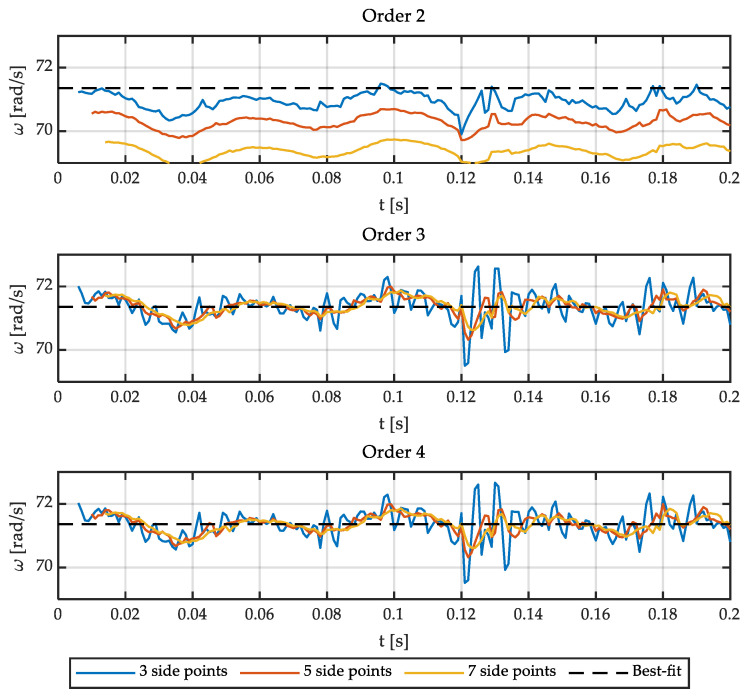
Angular speed fitted to polynomials of order 2, 3, and 4 with 3, 5, and 7 side points.

**Figure 4:**
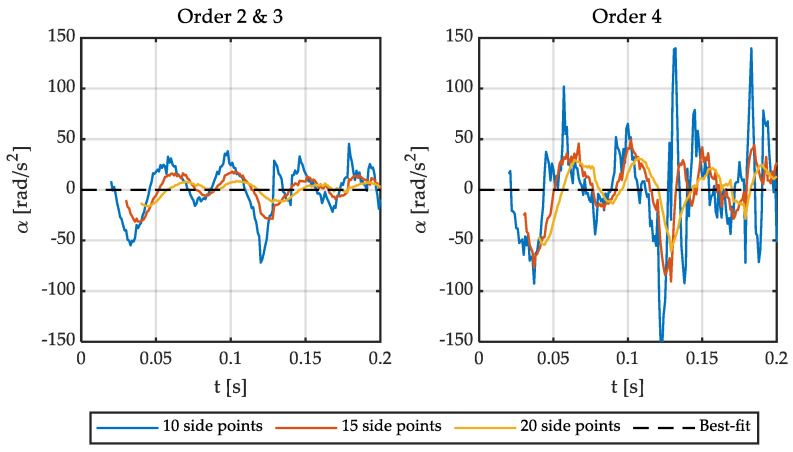
Angular acceleration fitted to polynomials of order 2, 3, and 4 with 10, 15, and 20 side points.

It is important to note that the delay is equal to the number of side points if the angular speed is calculated in real-time. Consequently, it is interesting to reduce the number of points used. On the other hand, the results obtained with a low-order polynomial (two or three) are satisfactory in the case of filtering a sinusoidal signal for some applications (i.e., estimating relative position). However, the order of the polynomial, as well as the number of points to be used, has to be determined if the goal is to accurately calculate the angular speed and acceleration. Therefore, the optimal polynomial order and the number of points have been studied for this application. Figure 1 shows different polynomials fitting the output signal of a Sin-Cos encoder. Figure 2 shows the errors of these fittings. Figure 3 and Figure 4 show the calculation of the angular speed and the angular acceleration respectively using different polynomials.

The procedure consists of the following. First, for comparison purposes, the data points are fitted by means of a sine, obtaining the fundamental parameters of the wave, namely: amplitude, frequency, and phase shift. In this case, the R-squared (R^2^) reached is greater than 0.999. These parameters determine the signal named “Fitted signal” or “Best-fit”. It is important to highlight the fact that this fitting operation cannot be performed in real-time. Despite being not appropriate for real system applications, it has been used to select the best filter configuration in this problem. Knowing the frequency of the sine wave, the angular speed of the wheel can be determined (*ω* = 2·π·f). In this case, there is no angular acceleration. These fitted values are the reference to estimate the errors of the process.

Then, the sine is fitted to polynomials of different orders and number of side points. This way, polynomials of order 2, 3, and 4; and 3, 5, and 7 side points are represented in Figure 1 and Figure 2. It is important to point out that matrix **g** has some interesting characteristics depending on the order of the polynomial. The convolution coefficients for the zeroth and second-time derivatives are identical in the case of polynomials of order 2 and 3, as well as 4 and 5. Similarly, in the case of the first and third-time derivative, the polynomials of orders 1 and 2 are the same, and those of orders 3 and 4 are equal too. Therefore, polynomial fitting of order 2 is not shown in Figure 1 and Figure 2 for the zeroth and second time derivative (it is identical to third order) and the fourth-order polynomial is not shown for the first time derivative (identical to third order). In the case of Figure 3, all orders of the polynomials are shown since time derivatives zero, one, and two are combined.

Figure 1 also shows that the delay increases as the number of side points does. In addition, it can be seen that a parabola is not able to fit the sine properly, slightly reducing the amplitude of the first derivative. As a result, the use of polynomials of at least order three for the calculation of angular speeds is required.

Finally, it can be seen that it is necessary to significantly increase the number of side points (and therefore the delay) in the case of polynomials of order 4 since the noise of these is considerably higher than those of order 3 (see Figure 1 and Figure 2).

In this work, it has been decided to use a cubic polynomial and 5 side points for the system under study. Once the number of side points has been decided, the delay can be precisely calculated. Moreover, the delay is the same whatever the frequency of the input signal. Furthermore, the delay of all time derivatives is identical provided that the same S-G filter parameters are used. These parameters are related to the computational cost, phase lag, and filtering effect. This way, the higher the number of side-points, the higher the delay but also the filtering effect. Conversely, the higher the order, the lower the filtering effect. The acquisition and processing of the analog signals are carried out at a frequency of 1 kHz. This way, the delay in the determination of the signals (position, speed, and acceleration) is 5 ms. This configuration allows an accurate calculation of speeds of up to 250 km/h for a medium-sized wheel (car, motorcycle, etc.). 

Table 1 shows the **g** matrix for the case previously mentioned. The table has been reproduced similarly as it was published in [45] so that only integers are written. Actually, these are fractional numbers, so it is necessary to divide each *g*_k_ by the divisor of its corresponding column. The procedure to calculate the **g** matrix for different cases can be found in [45,46], among others. In the case of using MATLAB^®^ (R2019b, Natick, MA, USA), the sgolay function directly returns the **g** matrix.

## 3. Results

The results of the described method are presented in this section. To do this, first, simulations with the most common methods used in commercial ABS encoders and Sin-Cos encoders were carried out. Then, the method proposed in this work is compared to the methods mentioned above. Next, real tests were conducted on the bench to evaluate the performance of these methods in a real system.

### 3.1. Simulations

**Figure 5:**
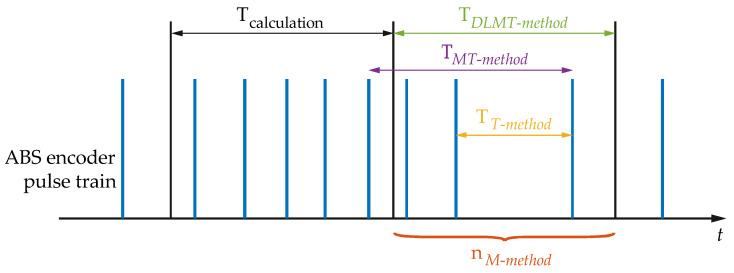
Measured variables for the four different methods mentioned.

Simulations were carried out to validate the proposed method, called *SinCos-method*. The ABS encoder used in the vehicle model MINI Cooper has been reproduced. This en-coder is the same one that will be later used in the real test. Figure 5 shows the measured variables for each of the four methods: *M-type*, *T-type*, *MT-type*, and *Divisionless-MT-type*.

Where T_calculation_ is the calculation period, T_MT-method_ is the time interval between the last pulse of the current period and the last pulse of the period in which at least one pulse was detected, T_T-method_ is the time interval between the last two pulses of the current interval, and n_M-method_ is the number of pulses in the current interval.

**Figure 6:**
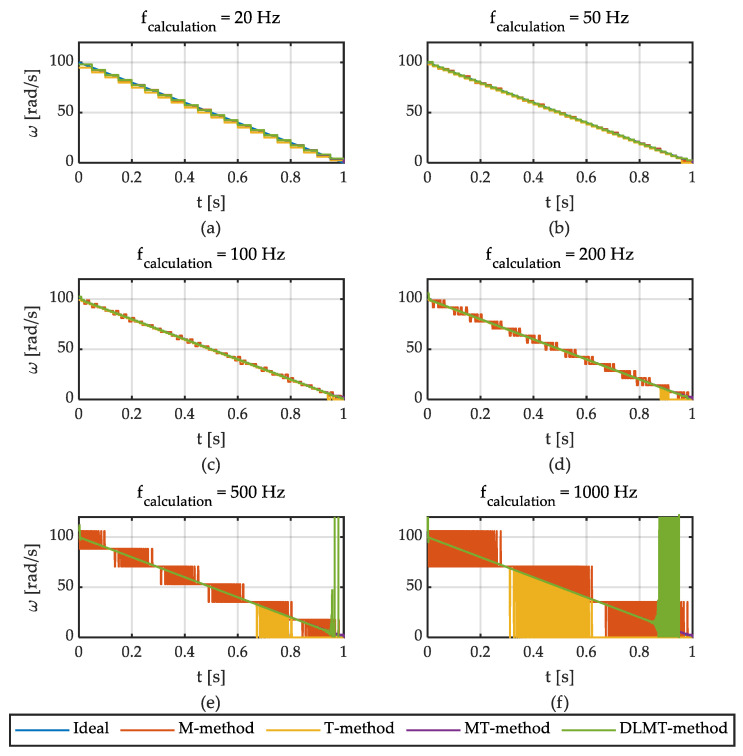
Angular speed estimation for different calculation frequencies.

First, the following analysis has been made to determine the calculation frequency (f_calculation_) of the angular speed from the ABS encoder. To do so, the angular speed given by the linear function *ω*(*t*) = 100 − 100 *t*, *t* ∈ [0, 1] has been simulated. The digital signal of the ABS encoder was sampled at f_sample,ABS_ = 1 MHz. The angular speed was calculated at six different frequencies, namely: f_calculation,ABS_ = 20, 50, 100, 200, 500, 1000 Hz. The angular speed has been determined for each of these six frequencies using four aforementioned methods. The term “Ideal” is used in the following figures to define the reference magnitude. The results are shown in Figure 6.

The following conclusions can be drawn from these results. The noise of the signals is small at low frequencies (Figure 6a,b) with greater delay as the calculation frequency decreases. *M* and *T* methods provide noisy signals for higher frequencies (Figure 6e,f). In both cases, the calculation frequency cannot be very high because a minimum number of pulses have to be counted within the calculation period. If no pulses are detected in the current period, the output of both methods (M and T) is equal to zero. 

It is important to note that the *T-method* performs well above a determined angular speed. Provided that at least two flanks of the encoder signal enter within a calculation period, the results yielded by this method are accurate (f_ABS_ ≥ 2 f_calculation_). On the other hand, the performance of the *MT-method* is fairly good regardless of the calculation frequency but for very low angular speeds. This is because this method resorts to previous calculation periods before the current one if no pulses enter in the current period. This way, it goes back to previous periods until it finds the last pulse that entered and the speed is obtained from data measured prior to the current calculation period. The disadvantage of the *MT-method* is that the computational cost is the highest of the three. Finally, the performance of the *Divisionless-MT-type* method is similar to the one provided by the *MT-type* method except for low speed, where high fluctuations are observed.

**Figure 7:**
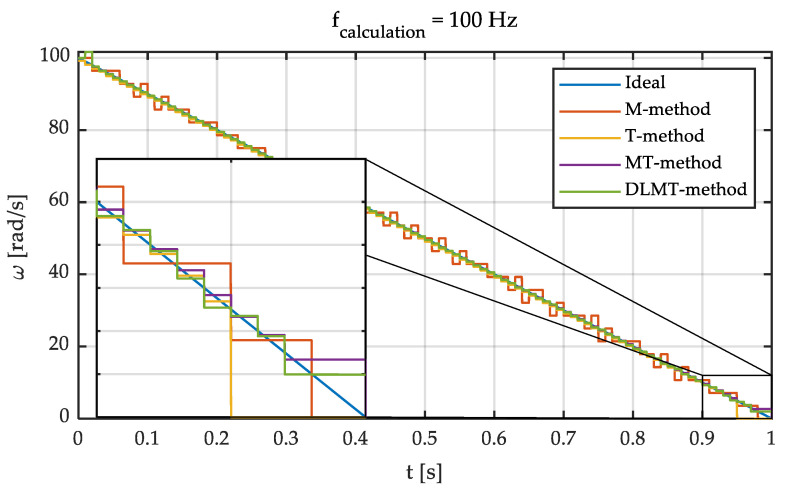
Simulation with different methods. d*ω*/d*t* = –100 rad/s^2^. *ω*_0_ = 100 rad/s.

Based on these previous results, an intermediate calculation frequency of 100 Hz has been chosen for the simulations and the experimental tests. This frequency is considered appropriate for traction and braking control applications since it shows a good balance between accuracy and measurement delay. Next, the comparison between the aforementioned methods is shown. To do so, simulations were carried out reproducing a braking process. Figure 7 shows the results obtained for an input angular speed given by the linear function *ω*(*t*) = 100−100 *t.* The following default parameters were set: f_sample,ABS_ = 1 MHz, f_calculation,ABS_ = 100 Hz, f_sample,Sin-Cos_ = f_calculation,Sin-Cos_ = 1 kHz. The number of pole pairs (i.e., pulses per revolution) of the ABS encoder is 176. 

It can be seen that the performance of the DLMT method is similar to the MT when the angular velocity is high. Conversely, the main disadvantage is that it is not able to provide adequate measures at low angular speeds. The following steps can be carried out to cope with this problem. The first one is to store the number of elapsed intervals without any input pulse. Next, it becomes necessary to make a division or store in memory the value of [(*n* − 1)·T_s_]^−1^, where *n* is the number of intervals that have elapsed without arriving pulses. Thus, these steps greatly increase the computational cost of this approach. Consequently, this method is discarded since a fast and accurate measurement of low angular speeds is required in traction control and braking algorithms, the MT being considered the most appropriate one for this type of applications. 

**Figure 8:**
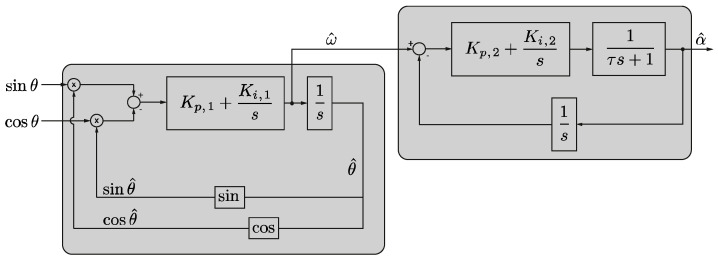
Dual-Phase-Locked Loop (PLL) scheme for estimation of angular speed and acceleration.

On the other hand, speed and angular acceleration with sinusoidal transducers can also be determined using a closed-loop dual-PLL configuration [38,39]. Figure 8 shows an outline of this approach. It consists of a first loop to determine angular velocity ($\hat{\omega }$) and position ($\hat{\theta }$) and a second loop, with a similar structure, to determine angular acceleration ($\hat{\alpha }$). This second loop includes a first-order low-pass filter with time constant (*τ*). Five parameters have to be set, namely: K_p,1_, K_i,1_, K_p,2_, K_i,2_, *τ*. These parameters should be tuned, taking into account the noise of the signals coming from the Sin-Cos encoder, the maximum allowable delay and the sampling frequency, among others.

**Figure 9:**
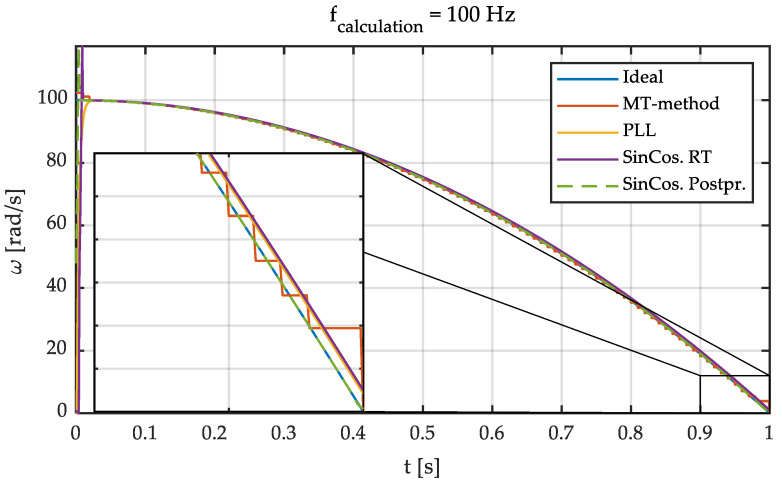
Simulation with different methods. d*ω*/d*t* = −200·*t* rad/s^2^. *ω*_0_ = 100 rad/s.

Next, a comparison between the MT-method, PLL, and the proposed method (SinCos. RT) is carried out. To do so, a braking process given by the parabolic function *ω*(*t*) = 100 − 100 *t*^2^ with the same default parameters used in the previous simulation was reproduced. Figure 9 shows the estimated angular speeds with the three methods.

As expected, the noise in the signals is greatly reduced with the selected calculation frequency of 100 Hz. Furthermore, the speed determined according to *MT*-*method* almost converges to the true value. Finally, it is observed that the PLL and *SinCos-methods* also perform satisfactorily. In this last case, the delay is constant being determined by the number of points chosen for the calculation of the speed. It has also been plotted the angular speed calculated using the SinCos-method replacing the dot product by a convolution operation. In this case, 15 side points have been selected and no delay exists between the calculated wheel speed and actual wheel speed. This demonstrates that this method can be used to obtain the angular speed for postprocessing applications. This output can also be considered as the reference wheel speed.

**Figure 10:**
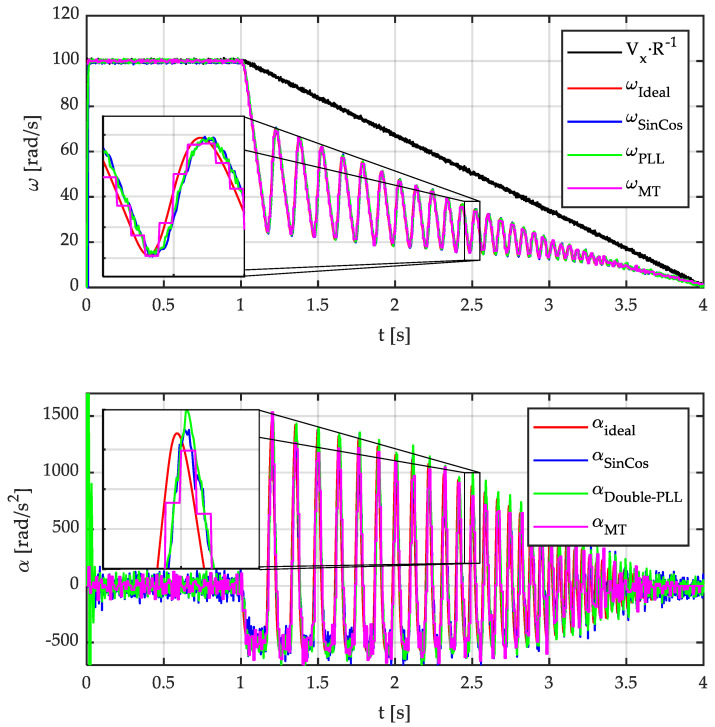
Simulated angular acceleration with noise. Sin-Cos amplitude: 4.5 V. Sin-Cos standard deviation noise: 6 mV.

Next, a braking process with an ABS is simulated. The initial linear velocity is 30 m/s. The wheel radius is 0.30 m. This way, angular speed is 100 rad/s for the first second. Then, a severe braking maneuver takes place. The following parameters were imposed: linear deceleration 10 m/s^2^, maximum angular deceleration 500 rad/s^2^, and maximum angular acceleration 1500 rad/s^2^. The slip ratio, that is, the ratio between peripheral wheel speed and vehicle speed, is kept between 20 and 80%. The vehicle fully stops after 3 s of braking. Figure 10 shows the angular velocity and acceleration (*α*) of the wheels. These magnitudes have been calculated using the three methods previously described. In the case of the acceleration estimation using the S-G Filter, as a second time derivative is required, it is recommended to increase the number of side points used. Therefore, a total of 6 side points have been considered for acceleration estimation. Figure 10 shows a simulation in which the sine and cosine signals have an amplitude of 4.5 V and white noise of *µ* = 0 and *σ* = 6 mV. These noise parameters have been considered to reproduce the measured noise of the real signals from the Sin Cos encoder.

To obtain angular acceleration from the incremental encoder, a simple two-point estimator has been considered (Equation (12)). Some other approaches could have been considered, such as Kalman filters [47], but at the expense of a high increase in the computational cost.

(12) $$\alpha_{k}^{MT}=\left(\omega_{k}^{MT}-\omega_{k-1}^{MT}\right)\cdot f,$$

With regards to the double PLL algorithm (Figure 8), five parameters have been properly tuned. The PI parameters of the first loop are K_p,1_ = 60 and K_i,1_ = 0.1. For the second loop, the value of these parameters are K_p,2_ = 400 and K_i,3_ = 0.1. The time constant of the first-order filter, *τ*, is set to 5 ms. It can also be noted that the contribution of the integral part of the PI is almost negligible. 

It can be seen that the PLL-based and S-G filter methods provide similar results. In both cases, the angular speed and acceleration match to real values adequately. Similar delays are obtained in both cases. On the contrary, the *MT-type method* provides discontinuous values due to discrete pulses from the incremental encoder and the calculation frequency. 

To evaluate the computational cost of each method, the time required to measure the speed in the previous simulation at the selected frequency of 100 Hz was recorded. An Intel(R) Core^TM^ i7-7700HQ CPU, 2.80 GHz, 16 GB RAM machine using MATLAB was used in this test. Simulations were repeated 1000 times and the average execution time was obtained. Results are listed in Table 2. It can be seen that the computation cost of the proposed methods is considerably lower compared to its competitors. 

### 3.2. Bench Tests

**Figure 11:**
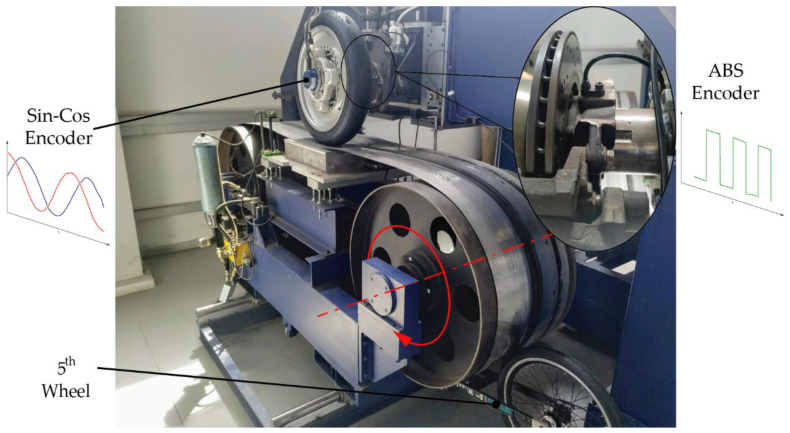
IMMa Flat track with speed sensors depicted.

For comparison purposes, the proposed *SinCos-method* and the *MT-method* were programmed in the IMMa (*Ingeniería Mecanica Malaga—Mechanical Engineering Malaga*) Flat Track tire test bench [47]. In this test bench, both the angular wheel speed (*ω*) and the linear belt speed (*V*_x_) can be measured (Figure 11). *M-method*, *T-method*, and *Divisionless-MT-type* were not included in this comparison because their performances are inferior compared to the *MT-method*, as it was shown in the previous subsection.

**Figure 12:**
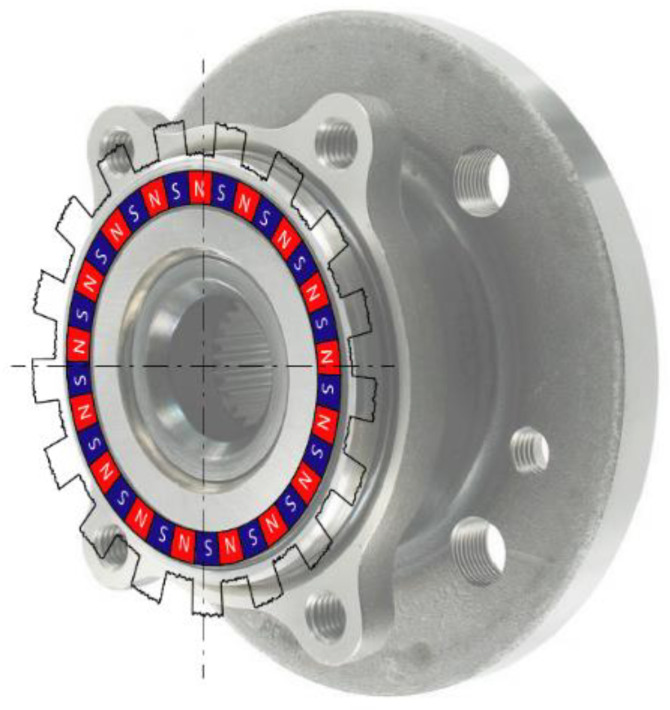
Incremental magnetic encoder, also known as ABS encoder (SKF VKBA 6634).

These comparative tests require the use of two sensors of different technologies: incremental magnetic encoder also called in the automotive field “ABS encoder” (Figure 12), and absolute encoder Sin-Cos. The former was placed beside the brake disk while the latter was already installed on a Kistler RoaDyn P625 wheel force transducer used in this test bench (Figure 11). Finally, A 5th wheel equipped with a high-end incremental magnetic encoder with 4000 pulses per revolution was used to measure the linear speed of the belt. 

A sbRIO-9637 Single-Board Controller by National Instruments™ was used to perform data acquisition. This device includes an FPGA and a real-time operating system. To avoid possible asymmetries in the output signal of the Hall effect sensor, the rising edges provided by the incremental encoder were used to measure the angular speed. Equation (13) was used to determine the angular speed from the ABS sensor:

(13) $$\omega_{\mathrm{ABS}}=\frac{2\;\pi \;x}{p\;T_{ABS}^{MT}},$$

where *ω*_ABS_ is the measured angular speed, *x* is the number of pulses that entered during $T_{ABS}^{MT}$, *p* is the number of pulses of the ABS encoder, $T_{ABS}^{MT}$ is the time gap between the last pulse of the previous calculation period and the last flank (see Figure 5). This time is measured by counting ticks within two pulses with a 1 MHz clock. The subscripts “ABS” and “Sin-Cos” are used to indicate that the signals come from the ABS encoder and Sin-Cos encoder respectively.

**Figure 13:**
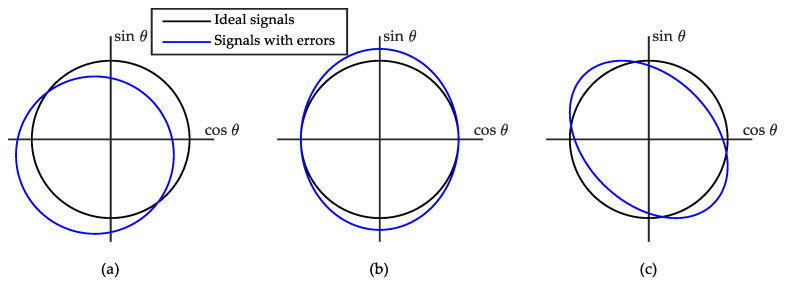
Three possible errors in the output signal of the Sin-Cos encoder. (**a**) Offset. (**b**) Different gains. (**c**) Adquisition delay.

Regarding the errors in the signals coming from the Sin-Cos encoder, three error sources can be identified [36], namely: offset between the magnet and the sensor (Figure 13a), different gain for each signal (Figure 13b), and delay in the acquisition of the signals (Figure 13c).

**Figure 14:**
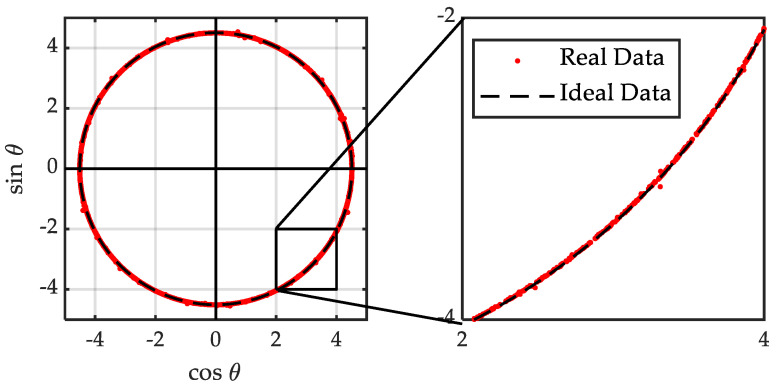
Sin-Cos sensor output signals.

None of the previously mentioned errors were observed when the Kistler RoaDyn P625 and sbRIO-9637 acquisition system were used (Figure 14). In any case, errors of type (**a**) and type (**b**) can be corrected by adding an offset and modifying the gain of one of the signals respectively. Both measurement corrections require a very low computational cost.

**Figure 15:**
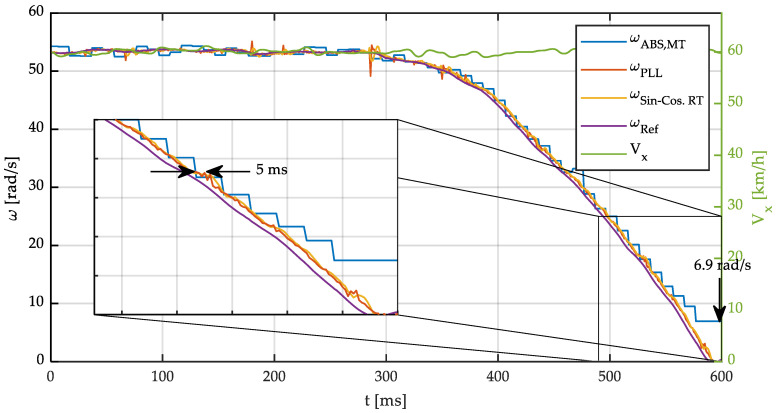
Wheel angular speed and linear speed vs. time. Vehicle speed 60 km/h.

**Figure 16:**
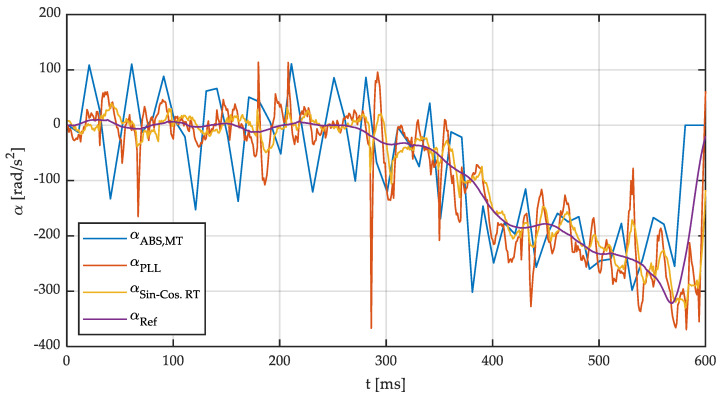
Wheel angular speed and linear speed vs. time. Vehicle speed 60 km/h.

The results of the measurement of the wheel’s angular speed while braking at different linear speeds are shown below. Figure 15 and Figure 16 show a braking process with an initial speed of 60 km/h (205/55 R15 tire). In these tests, the speed was kept constant for 0.3 s. Next, wheel angular speed was reduced by applying high pressure to the brake pads until the wheel locks. Belt speed was kept constant.

Figure 15 shows both the angular speed of the wheel and the linear speed of the belt while Figure 16 shows the angular acceleration of the wheel. Both magnitudes have been calculated through the three different methods previously described: MT, PLL, and SinCos. For verification purposes, the angular speed obtained using 15 side points and a polynomial of order 3 will be used as a reference in the experimental tests (ω_ref_ and α_ref_). This reference angular speed is obtained by postprocessing the measured signal with the parameters previously described. As shown previously in Figure 7 and Figure 9, the nondelayed angular speed and acceleration calculated by a convolution and more side points can be considered the reference angular speed since the error in the simulations is negligible. The following conclusions can be drawn from the observation of these results:

- The method proposed here is valid for both very low and high speeds. However, with an incremental encoder, even if the *MT-method* is used, errors are observed when measuring low speeds. This is of particular interest in the application of these sensors to brake control systems, where the wheels operate near the locking condition.
- Very low speeds and even wheel locking can be accurately calculated with the PLL and the proposed method. This is because Sin-Cos signals are continuous, as opposed to those of ABS encoders that are discontinuous.
- A low noise signal is obtained with the proposed method.
- If the signal from the ABS encoder is filtered to smooth the output, the filter will add a delay to this signal, which may be even longer than the delay of the signal from the Sin-Cos encoder.

It can be seen that wheel locking takes place at 600 ms. However, the *MT-method* does not detect this fact, producing a constant low value of angular speed in the last stage of the braking process. This behavior is due to the fact the angular speed is not updated because the sensor does not generate new pulses while the wheel is locked. On the other side, the proposed method provides an accurate measurement even at very low angular speed.

**Figure 17:**
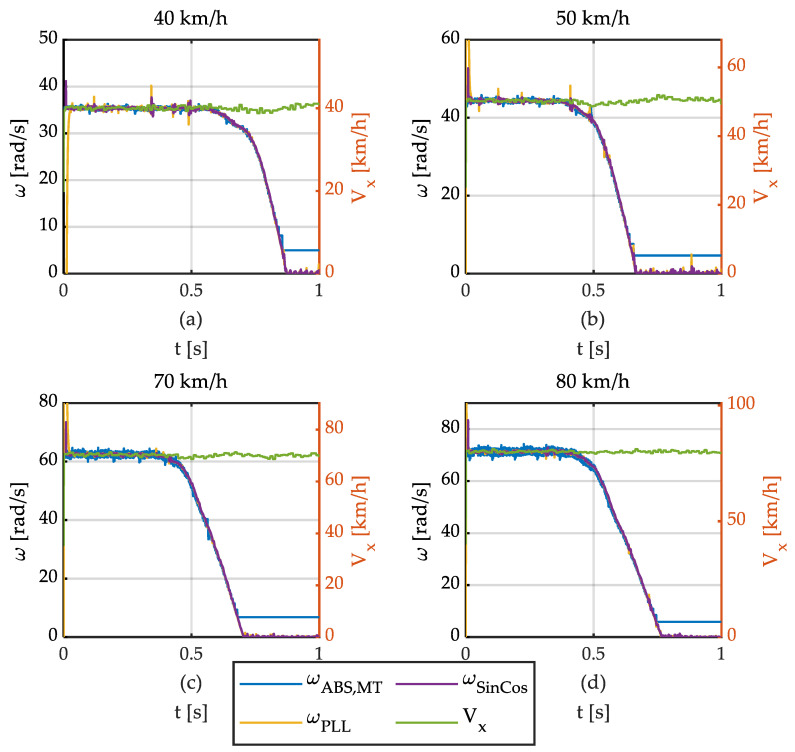
Wheel speed and linear speed vs. time. Different vehicle speeds. (**a**) 40 km/h. (**b**) 50 km/h. (**c**) 70 km/h. (**d**) 80 km/h.

Figure 17 shows braking tests at *V*_x_ = 40, 50, 70, and 80 km/h, showing similar results.

It can be seen that experimental results confirm the results obtained in simulations. This way, the MT method does not provide accurate measurements at low speed. Furthermore, highly noisy accelerations have been obtained. The PLL-based method and the S-G filter provide low-noise robust speed estimates. In addition, angular accelerations provided by both methods show a reasonably good correlation with real values.

## 4. Conclusions

Nowadays, most cars and motorcycles measure the angular speed of the wheels with incremental encoders. This work proposes a novel method for determining angular speeds using absolute encoders Sin-Cos. These are being increasingly used, especially in electric vehicles, although its implementation has not been extended to measure speeds but to determine the relative position of the rotor and stator in PMSM motors.

In this paper, the methods commonly used to determine wheel angular speeds have been described and a new method has been proposed. This method has been compared both in simulation and experimentally against commercial algorithms.

The advantages of the method presented are diverse. On the one hand, it makes it possible to measure very low speeds, close to wheel locking with a known and controlled delay. On the other hand, the computational cost is reduced, making it possible to be implemented in real-time applications. In addition, the noise from the signals is minimized through a least-squares fitting, providing accurate estimates of the position, speed, and angular acceleration signals.

As a summary and as an innovative contribution, it is important to note the following: the signals from a Sin-Cos encoder are time differentiated through the Savitzky–Golay filter. This way, the angular speed can be obtained in real-time with a reduced computational cost. Results show that improved estimates can be obtained with the proposed method. The performance of the Sin-Cos Method exceeds that of the most widely used commercial methods. Thus, it provides robust and accurate results from very low to high speeds. Current ABS encoders are not able to accurately measure or estimate wheel velocity in low speed conditions, which leads to poor wheel slip calculation. Consequently, the ABS is deactivated for speeds below 10–15 km/h, jeopardizing vehicle safety in braking maneuvers. The improved performance of the proposed method contributes to a better calculation of the wheel slip at low speed, thus enhancing ABS performance. This result shows that the use of this method can lead to the control improvement of ABS systems in vehicles. 

## Figures and Tables

**Table 1:** Matrix **g** for 5 side points and order 3.

g	Filter/ 0th Derivative	1st Derivative	2nd Derivative
g_k-10_	−36	300	30
g_k-9_	9	−294	12
g_k-8_	44	−532	−2
g_k-7_	69	−503	−12
g_k-6_	84	−296	−18
g_k-5_	89	0	−20
g_k-4_	84	296	−18
g_k-3_	69	503	−12
g_k-2_	44	532	−2
g_k-1_	9	294	12
g_k_	−36	−300	30
Divisor	429	5148	858

**Table 2:** Computational cost of the different methods.

Method	M	T	MT	DLMT	PLL	SinCos
Time (ms)	2.3531	4.8033	4.7263	3.4798	0.0251	0.0237

## Data Availability

The data presented in this study are available on request from the corresponding author.
